# Epidemiological and Clinical Characteristics of Acute Stroke in a Multi-Ethnic South Asian Population

**DOI:** 10.3390/neurolint17090140

**Published:** 2025-09-05

**Authors:** Kim H. Tran, Naveed Akhtar, Yahia Imam, Md Giass Uddin, Sujatha Joseph, Deborah Morgan, Blessy Babu, Ryan Ty Uy, Ashfaq Shuaib

**Affiliations:** 1 Department of Neurology, University of Alberta, 11350 83 Ave, Edmonton, AB T6G 2G3, Canada; 2 Department of Medicine, University of Manitoba, Winnipeg, MB R3Y 0A3, Canada; naveedakhtars@hotmail.com; 3 Neuroscience Institute, Hamad Medical Corporation, Doha 3050, Qatarruy1@hamad.qa (R.T.U.)

**Keywords:** South Asian, epidemiology, stroke outcomes, ischemic stroke

## Abstract

Objective: Stroke is one of the leading causes of death and disability worldwide. Compared to developed countries, the prognosis of stroke is less favourable in developing countries. The objective of this study is to identify inter-ethnic variation in risk profiles and stroke outcomes amongst Bangladeshi, Indian, Nepalese, Pakistani, and Sri Lankan expatriates living in Qatar. Methods: Data from the Qatar Stroke Registry were retrospectively analyzed from April 2014 to June 2025. A total of 8825 patients were included. The chi-square test was used to analyze sociodemographic variables, while the Kruskal–Wallis test was used to analyze continuous variables. Post hoc analysis was performed. Multivariate logistic regression and multivariate multiple regression were used to identify the predictors associated with poor clinical outcomes and mortality at 90 days. Results: Ischemic stroke was the predominant stroke type in all groups, with Nepalese patients presenting with stroke at a younger age, whilst Pakistanis tended to be older (*p* < 0.001). In terms of stroke outcomes, Nepalese patients had the highest proportion of a poor functional outcome at 90 days as well as NIHSS at discharge (*p* < 0.05). However, Bangladeshis had the highest proportion of mortality at 90 days compared to the other cohorts. Multivariable logistic regression revealed that undiagnosed dyslipidemia, Nepalese ethnicity, and moderate and severe NIHSS admission scores were independent predictors of a poor functional outcome at 90 days, whilst male sex and prior antidiabetic therapy were protective factors (*p* < 0.001). In terms of mortality at 90 days, only a severe NIHSS admission score (>10) was a significant predictor (*p* < 0.001). A severe NIHSS admission score was also the only predictive factor of mortality and poor functional outcome at 90 days (*p* < 0.05). Conclusions: There was a significant variation in stroke presentation and outcomes among South Asian subpopulations in Qatar, suggesting the importance of tailored public health strategies as a uniform approach to stroke care is insufficient for this diverse population.

## 1. Introduction

Stroke represents the second and third leading causes of death and disability worldwide [1], and in 2020, the incidence and number of deaths attributable to stroke were approximately 11.71 million and 7.08 million [2]. Ischemic stroke accounts for approximately 65% of stroke cases, whilst intracerebral hemorrhage and subarachnoid hemorrhage account for 29% and 6% of stroke cases, respectively [2]. Compared to developed countries, the prognosis of stroke is less favourable in developing countries due to a lack of relevant guidelines, limited resources, and a lack of awareness and treatment of risk factors associated with stroke [3,4,5].

Qatar is located on the northeastern border of the Arabian Peninsula, with a population of approximately 3 million, according to the World Health Organization. Overall, 15% of Qatar’s population is composed of native Qataris, whilst the remaining 85% consists of expatriates, the majority of whom are from the Indian subcontinent, i.e., India, Bangladesh, Pakistan, and Nepal [6]. Previous studies have shown that certain demographics and ethnic groups are at a greater risk for stroke due to their genetic profiles, dietary patterns, and lifestyle choices. For instance, compared to their White counterparts, South Asians have a higher prevalence of diabetes mellitus and cardiac diseases due to their cardiometabolic profile and dietary intake [7,8,9,10,11].

Within the South Asian subpopulation, the risk of vascular diseases, including stroke, varies among different ethnic groups due to their unique dietary habits and health profiles. For example, Indian populations tend to consume a high quantity of sweets, whilst Bangladeshi populations consume a high amount of deep-fried foods, both of which contribute differently to stroke-related risk factors [12]. Moreover, the prevalence of hypertension, a major risk factor for stroke, is also different: it is the highest amongst Nepalese (33.8%), followed by Indians (31.4%), Pakistanis (25%), Sri Lankans (20.9%), and Bangladeshis (17.9%) [13]. Coronary heart disease is also reportedly highest amongst Bangladeshis, followed by Pakistanis and Indians [14,15]. Bangladeshis also have the highest burden of diabetes, hyperlipidemia, and coronary heart disease amongst the South Asian subpopulations [16,17,18]. In the MASALA study, Reddy et al. reported that the cardiovascular risk factor profile of North and South Asian and Pakistani Americans differed [19].

While numerous studies have compared stroke outcomes amongst South Asians versus East Asians [20,21,22] or South Asians versus Whites, [11,23,24] there is a dearth of data comparing stroke outcomes amongst the South Asian subpopulations. The purpose of this study was to compare the prevalence of stroke risk factors and stroke outcomes in a multi-ethnic population comprising Sri Lankans, Indians, Nepalese, Pakistanis, and Bangladeshis. Our second aim was to identify the factors predictive of short-term prognosis post-stroke.

## 2. Methods

### 2.1. Study Population

Patient characteristics, including age, sex, nationality, medical comorbidities, and prior medication, were collected in the Stroke Registry. Data from the National Institute of Health Stroke Scale (NIHSS) score, neuroimaging data, and post-discharge disposition were entered into the registry. Ischemic stroke was diagnosed according to the WHO criteria [25] and stroke subtypes by the Trial of ORG 10172 in Acute Stroke Treatment (TOAST) criteria [26]. Modified Rankin scale (mRS) measurements were performed at discharge and at 90 days following the onset of symptoms. The patients were classified as favourable (mRS ≤ 0–2) or unfavourable (mRS 3–6) outcome. We used the dichotomized mRS scale as it is the most common method in use to evaluate recovery at 90 days [27].

Diabetes was diagnosed according to the American diabetes Association (ADA) and WHO recommendations [28] and included patients with a previous diagnosis of diabetes, on medication for diabetes, or an HbA1c of 6.5% or more, and the diagnosis of pre-diabetes was based on an HbA1c of 5.7–6.4%, as per the 2015 ADA clinical practice recommendations. Hypertension was defined as systolic blood pressure (BP) ≥ 140 mm Hg, a diastolic pressure ≥ 90 mm Hg, or on current treatment with antihypertensive drugs. Dyslipidemia was defined as low-density lipoprotein cholesterol level ≥ 3.62 mmol/L, high-density lipoprotein cholesterol level ≤ 1.03 mmol/L, triglycerides ≥ 1.69 mmol/L, or current treatment with a cholesterol-lowering drug.

### 2.2. Data Collection

Upon identification and confirmation of diagnosis using the International Classification of Disease, 10th edition, definitions (H34.1, I60.x, I61.x, I63.x, I64.x, G45.x, R29.818), the patients’ data were collected by trained stroke coordinators. The ethnicities of the patients were recorded at admission. This study was conducted in accordance with the Medical Research Centre (MRC) of Hamad Medical Corporation and received approval from the Institutional Review Board (MRC-01-18-1020) of Hamad Medical Corporation. All procedures adhered to ethical standards for clinical trials as mandated by the IRB and MRC. This study was approved by the IRB at the Medical Research Centre of Hamad Medical Corporation. All methods were carried out in accordance with the relevant guidelines and regulations of ICH and GCP. Written informed consent was waived by the IRB. Patient data were anonymized and securely managed by trained stroke coordinators to maintain confidentiality. The use of the Stroke Registry and compliance with the International Classification of Diseases (ICD-10) ensured standardized and ethical data collection.

### 2.3. Data Analysis and Statistics

Normality was assessed using the Kolmogorov–Smirnov test. Descriptive results for all continuous variables were reported as mean ± standard deviation (SD) for normally distributed data (using the independent samples *t*-test) or median with range for data with non-normal distributions (using the Kruskal–Wallis test). The Kruskal–Wallis test was performed along with Dunn’s post hoc test. The Pearson chi-square test and Fisher’s Exact Test were performed whenever appropriate to compare the proportion of all categorical variables between the groups. Chi-square pairwise comparisons with Bonferroni correction were also performed for categorical variables. In terms of identifying significant predictors of mortality at 90 days and poor functional outcome at 90 days (mRS of 3–6), we performed multivariable bivariate logistic regression, multivariable bivariate logistic regression by ethnicity stratification, and multivariable multiple regression. The latter was performed to identify the predictors associated with both mortality and poor functional outcome at 90 days. The results of regression models were reported using odds ratios (ORs) and 95% confidence intervals. A *p*-value of ≤0.05 (two-tailed) was considered significant. SPSS version 29.0.0.0 was used to perform the Kruskal–Wallis test, the Pearson chi-square test, Fisher’s Exact Test, and chi-square comparisons with Bonferroni adjustment. StataNow 18.5 was used for the multivariable analyses.

## 3. Results

### 3.1. Patient Characteristics

In our cohort of 8825 South Asian patients, 1948 (22.1%) were Bangladeshis, 3951 (44.8%) were Indians, 1126 (12.8%) were Nepalese, 1205 (13.7%) were Pakistanis, and 595 (6.7%) were Sri Lankans. There was a significant difference between the five groups in terms of age, with Nepalese being the youngest [median (IQR): 54.0 (47.0–61.0)] and Pakistanis being the oldest [63.0 (52.0–72.0)] (*p* < 0.001) (see Table 1). There was also a significant difference between the groups in terms of sex, with Nepalese having the highest proportion of men (97.8%). Post hoc analysis of the χ^2^ comparison revealed a significant association between male sex and Bangladeshi and Nepalese ethnicities. In contrast, there was a significant association between female sex and Indian and Pakistani ethnicities (see Table 2).

In terms of stroke subtypes, ischemic stroke was the predominant stroke type in all the groups (see Table 1). There was a significant association between intracerebral hemorrhage (ICH) and Bangladeshi and Nepalese ethnicities. When stroke type was stratified according to the TOAST classification, there was a significant difference between the groups (*p* < 0.001). However, this significance was lost after Bonferroni correction (see Table 2).

In terms of medical comorbidities, there was a significant difference between the groups in terms of diabetes, hypertension, dyslipidemia, and obesity (*p* < 0.05 for all), with Pakistanis having the highest proportion of these comorbidities (47.8%, 60.2%, 22.1%, and 33.4%, respectively). Post hoc analysis revealed a significant association between diabetes and Bangladeshi and Pakistani ethnicities and between hypertension, dyslipidemia, BMI (≥30), and Pakistani ethnicity. In contrast, there was a significant association between smokers and Bangladeshi ethnicity (*p* < 0.05 with Bonferroni correction). Amongst the vascular stroke risk factors, hypertension was the most frequent (ranging from 43.9% to 60.2%), followed by diabetes (ranging from 17.2% to 47.8%), smoking (ranging from 14.0% to 26.1%), obesity (ranging from 10.6% to 30.7%), and dyslipidemia (4.5% to 22.1%) (see Table 1).

### 3.2. Diagnosis

There were significant differences in the severity of stroke as measured by the NIHSS at admission (*p* < 0.001), with Nepalese presenting with more severe stroke, whilst Pakistanis and Indians presented with milder stroke. Ischemic stroke was similar in proportion between Bangladeshis (57.5%), Indians (60.0%), Nepalese (57.0%), and Sri Lankans (60.2%), but significantly lower in Pakistanis (52.2%). The proportion of intracerebral hemorrhage was highest amongst Nepalese (22.7%). Very few patients presented with central venous sinus thrombosis, and there was no significant difference between the five groups. Lastly, for transient ischemic attack, it was significantly high amongst Indians (9.1%; *p* < 0.05) compared to the other four ethnicities.

### 3.3. Outcome

For NIHSS at discharge, there was a significant difference between the groups, with Nepalese exhibiting the highest proportion of severe stroke (16.3%) (*p* < 0.05 after Bonferroni correction). Similarly, Nepalese had the highest proportion of poor functional outcomes at 90 days (mRS of 3–6) (35.5%) (*p* < 0.05 after Bonferroni correction). In terms of mortality at 90 days, it was significantly lowest amongst Indians (*p* < 0.05 after Bonferroni correction) but highest amongst Bangladeshis, albeit there was no significant association after Bonferroni correction.

### 3.4. Multivariable Analysis for Risk Factors Associated with Stroke Outcome

**Figure 1:**
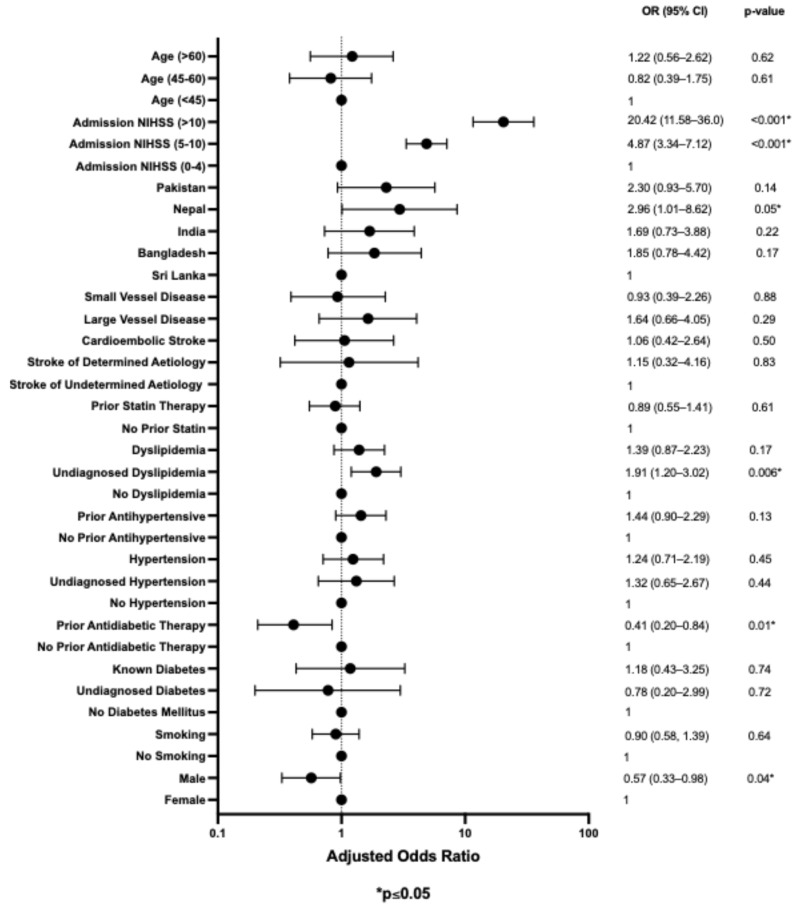
Multivariable bivariate logistic regression analysis of the risk factors associated with a higher mRS score (3–6) at 90 days.

**Figure 2:**
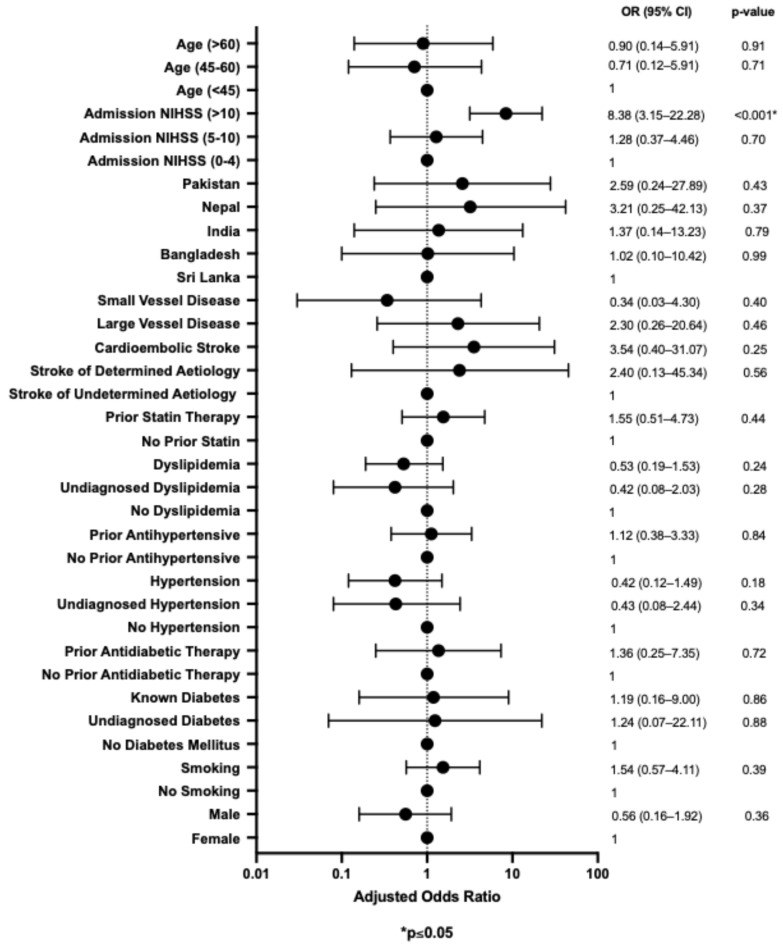
Multivariable bivariate logistic regression analysis of the risk factors associated with mortality at 90 days.

We developed a multivariable binary logistic regression model to identify significant independent factors associated with an mRS of 3–6 at 90 days and mortality at 90 days by adjusting for concomitant risk factors (see Figure 1 and Figure 2). The analysis revealed that moderate NIHSS (5–10) and severe NIHSS (>10) admission scores were significant predictors of a poor functional outcome at 90 days for all subpopulations [adjusted odds ratio (aOR) of 4.87, 95% confidence interval (CI): 3.34–7.12, *p* < 0.001; aOR 20.42, 95% CI: 11.58–36.0, *p* < 0.001, respectively]. Interestingly, Nepalese ethnicity and undiagnosed dyslipidemia were also independent predictors of a poor functional outcome at 90 days (aOR of 2.96 and 1.91, respectively). In contrast, male sex and prior antidiabetic therapy were protective against a poor functional outcome at 90 days (aOR 0.57 and 0.41, respectively).

In terms of mortality at 90 days, only a severe NIHSS admission score was a significant predictor (aOR of 8.38, 95% CI: 3.15–22.28, *p* < 0.001). Multivariable multiple regression revealed that a severe NIHSS admission score was the only significant predictor of mortality and poor functional outcome at 90 days (*p* < 0.001 for both; see Table 3).

### 3.5. Multivariable Analysis for Risk Factors Associated with Stroke Outcome by Ethnicity Stratification

For Bangladeshis, moderate and severe NIHSS admission scores were the only significant predictors of a poor functional outcome at 90 days (aOR 5.35 and 30.67, respectively). Small vessel disease was protective against a poor outcome at 90 days (aOR 0.36) (see Table 4). For Indians, moderate and severe NIHSS admission scores and known and undiagnosed diabetes were significant predictors of a poor outcome at 90 days. However, smoking and small vessel disease were protective (aOR 0.70 and aOR 0.50, respectively) (see Table 4). For Nepalese, moderate and severe NIHSS admission scores were significant predictors of a poor outcome at 90 days (aOR 7.35 and aOR 23.06, respectively) (see Table 4). For Pakistanis, moderate and severe NIHSS admission scores were significant predictors of a poor outcome at 90 days (aOR 3.16 and aOR 17.48). Interestingly, a stroke of determined etiology was protective against a poor functional outcome at 90 days for this cohort (aOR 0.27) (see Table 4). For Sri Lankans, moderate and severe NIHSS admission scores and known and undiagnosed hypertension were significant predictors of poor functional outcome at 90 days (see Table 4).

In terms of mortality at 90 days, a severe NIHSS admission score (>10) was a significant predictor for Bangladeshis (aOR of 19.75) and Indians (aOR 5.85) (see Table 5). However, for the latter, smoking and small vessel disease were protective factors against mortality at 90 days (aOR 0.18 and aOR 0.15, respectively). For Nepalese, a severe NIHSS admission score and known history of diabetes were independent predictors of mortality at 90 days (aOR 26.19 and aOR 53.5, respectively) (see Table 5). For Pakistanis, a severe NIHSS admission score and cardioembolic stroke were significant predictors of mortality at 90 days (aOR 15.59 and aOR 4.86, respectively) (see Table 5). For Sri Lankans, only a severe NIHSS admission score was a significant predictor of mortality at 90 days (aOR 14.00).

### 3.6. Excluding Stroke Mimics

When stroke mimics (defined as a clinical condition that resembles an acute stroke but is later confirmed to have a non-vascular etiology after diagnostic workup, i.e., seizures, migraine, functional neurological disorders) were removed from the analysis (n = 1877), similar observations were seen for age, gender, stroke type, TOAST, diabetes, hypertension, dyslipidemia, smoking, BMI, NIHSS at admission, mortality at 90 days, mRS at 90 days, and NIHSS at discharge (see Supplementary Table S1). The only difference was mRS at admission, where there was a significant positive association between Pakistani ethnicity and mRS at admission. Multivariable binary logistic regression revealed that moderate and severe NIHSS admission scores (aOR 4.86 and aOR 20.37), Nepalese ethnicity (aOR 3.05), and undiagnosed dyslipidemia (aOR 1.91) were independent predictors of a poor functional outcome at 90 days. Conversely, small vessel disease (aOR 0.94), prior antidiabetic therapy (aOR 0.41) and male sex (aOR 0.57) were protective against a poor functional outcome at 90 days (see Supplementary Figure S1). In terms of mortality at 90 days, only a severe NIHSS admission score (>10) was a significant predictor of mortality (aOR 8.37) (see Supplementary Figure S2). Similarly, the NIHSS admission score (>10) was the only significant predictor of both mortality and a poor functional outcome at 90 days (*p* < 0.001) (see Supplementary Table S3). When the multivariable binary analyses were stratified by ethnicity, similar outcomes were observed compared to the dataset that contained stroke mimics (see Supplementary Tables S4 and S5).

## 4. Discussion

South Asian individuals have unique genetics, cardiometabolic profiles, and dietary and lifestyle choices that increase their risk of stroke. In this 11-year retrospective analysis, we compared the clinical characteristics of acute stroke and outcomes amongst five major South Asian expatriate populations in Qatar. We found that Nepalese patients tend to be younger when presenting with stroke, whilst Pakistanis tend to be older (*p* < 0.001). Nepalese individuals presented with more severe stroke at admission (NIHSS > 10), whilst Pakistanis presented with milder stroke (*p* < 0.001). In terms of stroke diagnosis, the proportion of ischemic stroke was lowest amongst Nepalese (*p* < 0.001), while the proportion of intracerebral hemorrhage, CVST, and transient ischemic attacks (TIAs) were highest amongst Nepalese and Bangladeshis (*p* < 0.001), Nepalese and Pakistanis (*p* < 0.001), and Indians and Pakistanis (*p* < 0.001), respectively. In terms of stroke outcomes, Nepalese had the highest proportion of a poor functional outcome at 90 days and NIHSS at discharge (*p* < 0.001 for both). However, Bangladeshis had the highest proportion of mortality at 90 days compared to the other cohorts, whilst Indians had the lowest. Multivariable logistic regression also revealed that moderate and severe NIHSS admission scores (aOR 4.87 and aOR 20.42), undiagnosed dyslipidemia (aOR 1.91), and Nepalese ethnicity (aOR 2.96) were independent predictors of a poor functional outcome at 90 days, whilst male sex and prior antidiabetic therapy were protective factors (aOR 0.57 and aOR 0.41). In terms of mortality at 90 days, only a severe NIHSS admission score was a significant predictor (aOR 8.38). A severe NIHSS admission score was also the only variable that was a significant predictor of both mortality and poor outcome at 90 days (*p* < 0.001).

### 4.1. Stroke Outcomes and Prognostic Factors

In our study, Nepalese individuals had the worst functional outcomes at 90 days and were more likely to present with severe stroke at admission. Interestingly, they did not have the highest proportion of medical comorbidities, i.e., hypertension, diabetes, or dyslipidemia, compared to the other ethnicities, suggesting that the traditional risk factors associated with stroke might not be responsible for this cohort’s poor presentation and recovery post-stroke. Previous studies have reported that living in high altitudes (>2500 m) increases the risk of stroke through hypoxia-driven polycythemia, which increases the risk for hypercoagulation [29,30,31]. Cross-sectional analyses have also reported a significant association between living in high-altitude regions and stroke [32,33,34]. Indeed, Nepalese individuals tend to live at higher altitudes (from 59 m to 4080 m above sea level), which could potentially increase their risk of stroke and explain the younger age and severity of symptoms at presentation [35]. In a systematic review comprising 17 studies, Ortiz-Prado et al. reported that living at elevations between 1500 and 3500 m is protective against stroke, but at altitudes > 3500 m, there is extensive polycythemia, vascular stasis, and red blood cell and platelet adhesiveness, which increases the risk for thrombosis and stroke [36]. Our data are also consistent with previous studies that have shown that patients who live at higher altitudes tend to have an earlier age of diagnosis for ischemic stroke compared to patients from low altitudes [32,33,34,37].

### 4.2. Altitude, Hypoxia, and Stroke Subtypes

It is important to note that in our data, the proportion of ischemic stroke was lowest amongst Nepalese, whilst the proportion of intracerebral hemorrhage was highest. A potential explanation for this discrepancy is that in our dataset, we did not collect information on how these patients lived in Nepal and the altitude at which they lived. It is possible that more Nepalese patients live in the protective altitude range, i.e., 1500–3500 m, as posited by Ortiz-Prado et al.; therefore, there are fewer cases of ischemic strokes. Additional studies are needed to clarify this relationship. As for intracerebral hemorrhage, several studies have shown it is more common and more debilitating at higher altitudes due to the hypoxic environment, which leads to more severe hypertension and edema post-hemorrhage [38,39]. In addition, the hypoxic environment at high altitudes delays the body’s ability to repair damaged brain tissues [40]. At high altitudes, hematocrit is also increased, and studies have shown that high concentrations of hemoglobin can affect blood flow, which can exacerbate cerebral edema [32,41,42]. The diet in high altitude regions also consists of high-fat and high-salt foods, which could explain the high prevalence of intracerebral hemorrhage seen amongst Nepalese [43,44]. In a retrospective study conducted by Sha et al. [45], the authors reported that patients with central venous thrombosis from high altitudes had more severe clinical (headache and altered consciousness) and imaging manifestations (bleeding and venous infarction) compared to controls from low altitudes. This is consistent with our study, where we found a high proportion of CVST amongst Nepalese compared to the other nationalities, as these individuals reside in higher elevations.

### 4.3. Genetic, Occupational, and Environmental Influences

Our findings emphasize that traditional vascular risk factors alone do not fully explain the disparities in stroke presentation and outcomes observed among South Asian expatriate populations. In particular, the notably poor outcomes and severe stroke presentations among Nepalese patients—despite their lower prevalence of common comorbidities such as hypertension and diabetes—suggest that other, less conventional contributors may be at play. There is growing interest in exploring genetic predisposition, molecular pathways, and epigenetic modifications that may underlie susceptibility to stroke in specific ethnic groups. Recent work has shown that certain South Asian subpopulations may carry unique genetic polymorphisms that predispose them to higher cardiovascular risk, independent of traditional markers [8,14]. Investigating these biological factors may pave the way for precision medicine strategies in stroke prevention and recovery. Additionally, the unique environmental and occupational context of expatriate life in Qatar—particularly in Doha—should not be overlooked. Many Nepalese and Bangladeshi individuals are employed in physically demanding, outdoor “blue-collar” jobs, such as construction, cleaning, or transportation, which expose them to extreme temperatures, air pollution, and chronic dehydration. There is increasing evidence that exposure to particulate matter (PM2.5) and heat stress significantly raises the risk of cardiovascular and cerebrovascular events [42,46]. Studies from the Gulf region have specifically linked elevated PM2.5 levels with increased rates of stroke hospitalization and mortality [47]. Furthermore, the physically strenuous work conditions and long hours, often combined with limited access to preventive healthcare, may accelerate vascular wear and tear in these individuals. This could help explain why stroke affects them at a younger age and with greater severity, as seen in our Nepalese cohort.

Given these findings, future research should aim to incorporate occupational profiles, environmental exposures, and residency duration in host countries into risk stratification models. Longitudinal studies examining the interaction between genetic susceptibility, air quality, and workplace stressors will be essential to develop more effective, equitable stroke prevention strategies for South Asian expatriates in the Gulf.

### 4.4. Mortality and TIA Patterns Across South Asian Subgroups

Moreover, our study is partially consistent with previous research that has examined the proportion of mortality and TIAs in South Asians. Venketasubramanian previously reported that amongst South Asians, the mortality rate was highest in Bangladeshis and Pakistanis and lowest in Sri Lankans, while the proportion of TIA was highest in Sri Lankans [21]. In our study, the mortality rate was highest in Pakistanis (5.8%) and Bangladeshis (5.7%) but lowest amongst Indians (3.6%) (when stroke mimics were removed from the analysis). In contrast, Pakistanis had the highest proportion of TIAs. A potential explanation for this discrepancy is that in Venketasubramanian’s paper, the proportion of TIA is missing for several countries (Bangladesh, Bhutan, India, and Pakistan), providing an incomplete picture of the proportion of TIAs amongst these ethnicities, thus preventing a meaningful comparison. Another explanation for the low mortality rate observed amongst Indians compared to Pakistanis and Bangladeshis could be stronger public health initiatives in stroke prevention, improved health coverage, and improved success in tobacco control in India compared to the latter countries [48]. Pakistanis also have insufficient awareness of stroke and stroke-related risk factors [49]. Interestingly, in our cohort of Indian patients, smoking was associated with lower 90-day mortality and better functional outcomes, a pattern reminiscent of the so-called smoker’s paradox observed in earlier reports [50]. However, a comprehensive meta-analysis of 21 studies demonstrated that, after full adjustment for confounders, smoking had no protective effect on ischemic stroke prognosis (adjusted OR for poor outcome ≈ 0.96, 95% CI 0.77–1.21), and smokers presented nearly 10 years earlier than nonsmokers at stroke onset [51]. More recent large-scale investigations—including a 2024 registry from Japan—found no significant benefit of smoking on outcomes after reperfusion therapy, even with propensity-score adjustment [52]. Thus, the apparent association in our Indian subgroup is likely driven by younger age, less severe stroke phenotypes, such as small vessel disease, or treatment-related confounders, rather than any true biological protection from tobacco use. Consequently, this finding must be interpreted with caution and should not be misconstrued as evidence that smoking is beneficial.

### 4.5. Cultural, Dietary, and Lifestyle Variations Among South Asians

While previous studies have focused on how stroke risk, incidence, and mortality rates vary between ethnicities [53,54,55,56], our study is unique in that it highlights how these variables can also vary between countries/nationalities that share a common lineage. Indeed, the modifiable risk factors associated with stroke can vary between these populations. For example, the prevalence of smoking is highest in the Maldives, followed by Nepal, India, Afghanistan, and Pakistan, suggesting people’s preferences for smoking differ across countries [57]. Dietary choices also vary substantially depending on the person’s religious/cultural affiliations. For example, Northern Indians consume a delicacy known as ghee (clarified butter), which has been associated with coronary artery disease, although not all South Asian populations consume it [58,59]. Bhotla et al. also reported that a majority of South Asians are vegetarians due to cultural and religious obligations, and this diet lacks various micronutrients, such as cobalamin (vitamin B12), vitamin D, and omega-3 fatty acids, which increases the risk for cardiovascular diseases and stroke [60]. However, not all South Asians are vegetarians. For example, meat plays an important role in Pakistani cuisine compared to other South Asian cultures, and Pakistanis, on average, consume more meat than Indians [61]. Thus, future guidelines/protocols for stroke prevention should take into account the heterogeneous vascular profiles of these South Asian subpopulations instead of utilizing a homogeneous approach.

In terms of the risk factors associated with a poor clinical outcome and mortality at 90 days, they are consistent with our previous studies [5,62]. However, Nepalese ethnicity is a novel predictor in this model, highlighting the need for more proactive management within this population.

### 4.6. Strengths and Limitations

This study offers several notable strengths. It represents one of the largest multi-ethnic analyses of South Asian expatriates with stroke in the Gulf region, drawing on a national registry with systematically collected clinical data, robust stroke classification using TOAST criteria, and standardized functional outcome assessment at 90 days. Importantly, this study disaggregates South Asian populations by nationality, revealing within-group disparities that are often masked in broader ethnic analyses.

However, some limitations merit acknowledgment. First, the lack of data on duration of residency in Qatar, occupational roles, and socioeconomic status limits our ability to assess the impact of acculturation and work-related exposures on stroke outcomes. Second, we did not collect data on dietary habits, environmental exposures (e.g., air pollution), or genetic markers, all of which may influence stroke risk and recovery. Third, this study did not differentiate between ischemic stroke subtypes using advanced imaging modalities or biomarker profiles, which could help refine etiological understanding in younger patients with severe strokes. Finally, as a retrospective analysis, our findings are hypothesis-generating and limited by potential residual confounding.

Future studies should adopt a prospective, multi-level approach incorporating genetic profiling, epigenetic markers, occupational categories, and environmental stressors, including pollution and temperature exposure. Stratifying by type of employment—such as indoor administrative roles versus outdoor labour-intensive jobs—could shed light on whether the occupational environment contributes to stroke severity, especially in younger Nepalese and Bangladeshi men. Moreover, qualitative work exploring health-seeking behaviour, awareness, and access barriers in these communities may uncover modifiable factors amenable to public health intervention. Ultimately, a precision stroke prevention model, informed by both biological and contextual data, is essential to address the health needs of diverse South Asian populations living abroad.

## 5. Conclusions

This study offers critical insight into the heterogeneity in stroke presentation and outcomes among South Asian expatriate populations in Qatar, underscoring the importance of disaggregating ethnic data within this broadly defined group. Notably, Nepalese patients exhibited the most severe clinical presentations and poorest outcomes at 90 days, despite having fewer traditional vascular risk factors, suggesting the influence of non-traditional factors such as altitude-related physiology. In contrast, Pakistanis had a milder stroke presentation but showed the highest rates of TIAs and a higher mortality rate alongside Bangladeshis. These findings highlight how factors like altitude, dietary habits, and healthcare access significantly shape stroke risk and recovery, even among populations with shared ancestry.

The identification of Nepalese ethnicity as an independent predictor of poor functional outcome at 90 days is a novel and important contribution, indicating the need for tailored public health strategies and early interventions in this subgroup. Furthermore, our findings reinforce that a one-size-fits-all approach to stroke prevention and management is inadequate for South Asian populations. Future research should aim to clarify the roles of high-altitude residence, duration of expatriation, and lifestyle factors in shaping stroke risk and outcomes, particularly for underrepresented groups like the Nepalese. Addressing these gaps is essential to developing more equitable and effective stroke care protocols for South Asians in both native and expatriate contexts.

## Figures and Tables

**Table 1:** Baseline characteristics with stroke mimics.

	Overall	Bangladesh	India	Nepal	Pakistan	Sri Lanka	Overall Significance
**Total patients**	8825	1948	3951	1126	1205	595	
**Age**	55.0 (47.0–63.0)	54.0 (47.0–61.0)	56.0 (48.0–63.0)	49.0 (44.0–55.0)	63.0 (52.0–72.0)	56.0 (48.0–62.0)	<0.001 *
<45	1565 (17.7)	364 (18.7)	631 (16.0)	325 (28.9)	163 (13.5)	82 (13.8)	<0.001
45–60	4481 (50.8)	1035 (53.1)	2035 (51.5)	737 (65.5)	337 (28.0)	337 (56.6)	
>60	2779 (31.5)	549 (28.2)	1285 (32.5)	64 (5.7)	705 (58.5)	176 (29.6)	
**Sex**							<0.001
Male	8002 (90.7)	1889 (97.0)	3529 (89.3)	1101 (97.8)	251 (20.8)	529 (88.9)	
Female	823 (9.3)	59 (3.0)	422 (10.7)	25 (2.2)	954 (79.2)	66 (11.1)	
**Stroke Type**							<0.001
Central Venous Sinus Thrombosis	127 (1.4)	23 (1.2)	54 (1.4)	24 (2.1)	19 (1.6)	7 (1.2)	
Intracerebral Hemorrhage	1101 (12.5)	295 (15.1)	392 (9.9)	256 (22.7)	90 (7.5)	68 (11.4)	
Ischemic Stroke	5070 (57.5)	1168 (60.0)	2273 (57.5)	642 (57.0)	629 (52.2)	358 (60.2)	
Transient Ischemic Attack	650 (7.4)	98 (5.0)	361 (9.1)	54 (4.8)	104 (8.6)	33 (5.5)	
Stroke Mimic	1877 (21.3)	364 (18.7)	871 (22.0)	150 (13.3)	363 (30.1)	129 (21.7)	
**TOAST**							0.04
Small Vessel Disease	2400 (46.3)	573 (48.4)	1070 (46.1)	321 (48.3)	265 (40.9)	171 (47.0)	
Large Vessel Disease	1128 (21.8)	256 (21.6)	505 (21.8)	138 (20.8)	146 (22.5)	83 (22.8)	
Cardioembolic	952 (18.4)	219 (18.5)	425 (18.3)	105 (15.8)	142 (21.9)	61 (16.8)	
Determined Etiology	448 (8.6)	77 (6.5)	216 (9.3)	58 (8.7)	63 (9.7)	34 (9.3)	
Undetermined Etiology	255 (4.9)	60 (5.1)	105 (4.5)	43 (6.5)	32 (4.9)	15 (2.5)	
**Medical Comorbidities**							
Diabetes	3241 (36.7)	784 (40.2)	1481 (37.5)	194 (17.2)	576 (47.8)	206 (34.6)	<0.001
Hypertension	4605 (52.2)	1040 (53.4)	2042 (51.7)	494 (43.9)	725 (60.2)	304 (51.1)	<0.001
Dyslipidemia	1146 (13.0)	243 (12.5)	517 (13.1)	50 (4.5)	266 (22.1)	70 (11.8)	<0.001
Smoking	1849 (21.0)	506 (26.1)	837 (21.3)	202 (18.0)	168 (14.0)	136 (23.0)	<0.001
Obesity (BMI ≥ 30)	1639 (18.8)	233 (12.1)	727 (18.6)	188 (16.8)	396 (33.4)	95 (16.1)	<0.001
**Management**							
Thrombolysis	649 (7.4)	130 (6.7)	307 (7.8)	86 (7.6)	87 (7.2)	39 (6.6)	0.55
Thrombectomy	240 (2.7)	46 (2.4)	110 (2.8)	44 (3.9)	22 (1.8)	18 (3.0)	0.03
**NIHSS Admission**							<0.001
Mild Stroke (0–4)	5718 (65.1)	1206 (62.3)	2676 (68.0)	600 (53.4)	848 (70.8)	388 (65.8)	
Moderate Stroke (5–10)	1659 (18.9)	377 (19.5)	732 (18.6)	245 (21.8)	197 (16.4)	108 (18.3)	
Severe Stroke (≥11)	1403 (16.0)	353 (18.2)	525 (13.3)	278 (24.8)	153 (12.8)	94 (15.9)	
**mRS at Admission**							<0.001
0–2	8617 (97.7)	1917 (98.4)	3877 (98.2)	1125 (99.9)	1108 (92.0)	590 (99.2)	
3–6	207 (2.3)	31 (1.6)	73 (1.8)	1 (0.1)	97 (8.0)	5 (0.8)	
**NIHSS at Discharge**							<0.001
Mild Stroke (0–4)	5341 (73.5)	1125 (68.9)	2497 (76.0)	579 (63.2)	785 (80.8)	355 (76.0)	
Moderate Stroke (5–10)	1143 (15.7)	309 (18.9)	472 (14.4)	188 (20.5)	104 (10.7)	70 (15.0)	
Severe Stroke (≥11)	786 (10.8)	198 (12.1)	315 (9.6)	149 (16.3)	82 (8.4)	42 (9.0)	
**mRS at 90 Days**							<0.001
0–2	3794 (71.3)	821 (69.2)	1766 (74.4)	454 (64.5)	488 (70.2)	265 (73.2)	
3–6	1527 (28.7)	365 (30.8)	608 (25.6)	250 (35.5)	207 (29.8)	97 (26.8)	
**Mortality at 90 Days**							0.02
No	5080 (95.5)	1119 (94.4)	2291 (96.5)	671 (95.3)	657 (94.5)	342 (94.5)	
Yes	241 (4.5)	67 (5.6)	83 (3.5)	33 (4.7)	38 (5.5)	20 (5.5)	

* Bonferroni correction was applied using Dunn’s test. All other *p*-values reported are unadjusted *p*-values derived from the χ^2^ test.

**Table 2:** Values reported are χ^2^ adjusted residuals. * *p* ≤ 0.05 with Bonferroni correction.

	Bangladesh	India	Nepal	Pakistan	Sri Lanka
**Age**					
<45	1.25	−3.90 *	10.47 *	−4.11 *	−2.61
45–60	2.36	1.23	10.55 *	−17.04 *	2.96 *
>60	−3.56 *	1.88	−19.96 *	21.73 *	−1.04
**Gender**					
Male	10.83 *	−3.94 *	8.78 *	−14.78 *	−1.53
Female	−10.83 *	3.94 *	−8.78 *	14.78 *	1.53
**TOAST**					
Small Vessel Disease	1.61	−0.27	1.09	−2.95	0.27
Large Vessel Disease	−0.15	−0.01	−0.68	0.51	0.50
Cardioembolic	0.11	−0.09	−1.84	2.49	−0.82
Determined Etiology	−2.99	1.53	0.08	1.04	0.49
Undetermined Etiology	0.26	−1.19	1.97	0.02	−0.73
**Stroke Type**					
Central Venous Sinus Thrombosis	−1.08	−0.51	2.09	0.42	−0.56
Intracerebral Hemorrhage	4.04 *	−6.54 *	11.15 *	−5.66 *	−0.80
Ischemic Stroke	2.54	0.14	−0.32	−3.97 *	1.39
Transient Ischemic Attack	−4.47 *	5.74 *	−3.53 *	1.81	−1.76
Stroke Mimic	−3.16*	1.60	−6.98 *	8.08 *	0.25
**Medical Comorbidities**					
**Diabetes**					
Known	3.65 *	1.33	−14.53 *	8.58 *	−1.10
Undiagnosed	2.15	0.16	1.33	−4.75 *	0.85
No	−4.84 *	−1.38	13.23 *	−5.41 *	0.55
**Hypertension**					
Known	1.23	−0.85	−5.98 *	5.97 *	−0.55
Undiagnosed	−0.52	0.03	6.78 *	−5.01 *	−1.37
No	−0.92	0.87	1.42	−2.68	1.57
**Dyslipidemia**					
Known	−0.78	0.24	−9.11 *	10.09 *	−0.90
Undiagnosed	0.88	2.08	0.93	−7.82 *	3.90 *
No	−0.24	−2.02	5.48 *	−0.01	−2.85
**Smoking**					
Yes	6.15 *	−0.48	2.63	−6.45 *	−1.20
No	−6.15 *	0.48	−2.63	6.45 *	1.20
**BMI**					
<30	8.48 *	0.38	1.81	−13.86 *	1.74
≥30	−8.48 *	−0.38	−1.81	13.86 *	−1.74
**mRS at Admission**					
0–2	−2.49	−2.78	−5.36 *	14.08 *	−2.51
3–6	2.49	2.78	5.36 *	−14.08 *	2.51
**NIHSS Admission**					
0–4	−2.96 *	5.16 *	−8.81 *	4.42 *	0.34
5–10	0.74	−0.61	2.68	−2.33	−0.38
≥11	3.07 *	−6.06 *	8.59 *	−3.26 *	−0.03
**Mortality at 90 Days**					
Yes	2.10	−3.25 *	0.22	1.28	0.94
No	−2.10	3.25*	−0.22	−1.28	−0.94
**mRS at 90 Days**					
0–2	1.79	4.47 *	−4.29 *	−0.68	0.83
3–6	−1.79	−4.47 *	4.29 *	0.68	−0.83
**NIHSS at Discharge**					
0–4	−4.71 *	4.50 *	−7.52 *	5.59 *	1.29
5–10	4.05 *	−2.87	4.27 *	−4.61 *	−0.45
≥11	1.95	−3.04 *	5.69 *	−2.55	−1.31

**Table 3:** Multivariable multiple regression for mortality at 90 days and mRS at 90 days.

mRS at 90 Days	Coef.	Std. Err.	t-Value	95% CI	*p*-Value
**Age**					
45–60	−0.021	0.059	−0.35	(−0.14, 0.096)	0.72
>60	0.037	0.061	0.61	(−0.08, 0.157)	0.55
**Gender**					
Male	−0.098	0.046	−2.11	(−0.19, −0.007)	0.04
**Ethnicity**					
India	−0.016	0.034	-0.48	(−0.08, 0.051)	0.64
Nepal	0.077	0.066	1.17	(−0.05, 0.206)	0.24
Pakistan	0.034	0.046	0.75	(−0.06, 0.124)	0.46
Sri Lanka	−0.081	0.061	−1.33	(−0.20, 0.038)	0.18
**Smoking**					
Yes	−0.015	0.033	−0.45	(−0.08, 0.050)	0.65
**Diabetes**					
Known	0.045	0.082	0.55	(−0.12, 0.205)	0.58
Undiagnosed	−0.019	0.103	−0.19	(−0.22, 0.183)	0.85
**Hypertension**					
Known	0.025	0.042	0.60	(−0.06, 0.108)	0.55
Undiagnosed					0.53
**Dyslipidemia**					
Known	0.056	0.037	1.50	(−0.02, 0.129)	0.14
Undiagnosed	0.103	0.037	2.80	(0.03, 0.175)	0.005
**NIHSS Admission**					
5–10	0.293	0.033	8.87	(0.23, 0.357)	<0.001
11–40	0.603	0.045	13.5	(0.52, 0.691)	<0.001 *
**Diabetic Therapy**					
Yes	−0.143	0.058	−2.47	(−0.26, −0.029)	0.014
**Statin**					
Yes	−0.016	0.037	−0.44	(−0.09, 0.057)	0.66
**Antihypertensive**					
Yes	0.059	0.037	1.61	(−0.01, 0.131)	0.11
**Mortality at 90 Days**	**Coef.**	**Std. Err.**	**t-Value**	**95% CI**	***p*** **-Value**
**Age**					
45–60	−0.006	0.026	−0.23	(−0.06, 0.045)	0.82
>60	0.002	0.027	0.08	(−0.05, 0.054)	0.94
**Gender**					
Male	−0.025	0.020	−1.23	(−0.06, 0.015)	0.22
**Ethnicity**					
India	0.005	0.015	0.34	(−0.02, 0.034)	0.73
Nepal	0.041	0.029	1.42	(−0.02, 0.097)	0.16
Pakistan	0.027	0.020	1.38	(−0.01, 0.066)	0.17
Sri Lanka	0.005	0.026	0.19	(−0.05, 0.057)	0.85
**Smoking**					
Yes	0.012	0.015	0.85	(−0.02, 0.041)	0.40
**Diabetes**					
Known	−0.007	0.035	−0.19	(−0.08, 0.063)	0.85
Undiagnosed	0.009	0.045	0.19	(−0.08, 0.097)	0.85
**Hypertension**					
Known	−0.016	0.018	−0.87	(−0.05, 0.020)	0.38
Undiagnosed	−0.021	0.024	−0.88	(−0.07, 0.026)	0.38
**Dyslipidemia**					
Known	−0.019	0.016	−1.15	(−0.05, 0.013)	0.25
Undiagnosed	−0.013	0.016	−0.82	(−0.04, 0.018)	0.41
**NIHSS Admission**					
5–10	0.001	0.014	0.10	(−0.03, 0.030)	0.92
11–40	0.125	0.019	6.44	(0.09, 0.164)	<0.001 *
**Diabetic Therapy**					
Yes	0.019	0.025	0.77	(−0.03, 0.069)	0.44
**Statin**					
Yes	0.016	0.016	0.96	(−0.02, 0.048)	0.34
**Antihypertensive**					
Yes	0.001	0.016	0.08	(−0.03, 0.033)	0.94
**mRS at 90 days**					
**Obs**			**R-sq**		
879			0.272		
**Parms**			**F**		
24			13.92		
**Root-mean-square deviation**			**Prob > F**		
0.396			<0.001		
**Mortality at 90 days**					
**Obs**			**R-sq**		
879			0.091		
**Parms**			**F**		
24			3.70		
**Root-mean-square deviation**			**Prob > F**		
0.173			<0.001		

* *p* ≤ 0.05.

**Table 4:** (**a**) Multivariate bivariate logistic regression for factors associated with a poor functional outcome (mRS of 3–6) at 90 days for Bangladeshis. (**b**) Multivariate bivariate logistic regression for factors associated with a poor functional outcome (mRS of 3–6) at 90 days for Indians. (**c**) Multivariate bivariate logistic regression for factors associated with a poor functional outcome (mRS of 3–6) at 90 days for Nepalese. (**d**) Multivariate bivariate logistic regression for factors associated with a poor functional outcome (mRS of 3–6) at 90 days for Pakistanis. (**e**) Multivariate bivariate logistic regression for factors associated with a poor functional outcome (mRS of 3–6) at 90 days for Sri Lankans.

Variable	aOR	*p*-Value	95% CI
(a)			
**Age**			
>60	0.73	0.34	0.38, 1.40
45–60	0.75	0.35	0.42, 1.36
<45	-	-	-
**Gender**			
Male	1.23	0.76	0.34, 4.40
Female	-	-	-
**Smoking**			
Yes	1.21	0.36	0.81, 1.82
**NIHSS Admission**			
5–10	5.35	<0.001 *	3.49, 8.22
11–40	30.67	<0.001 *	17.60, 53.42
**Diabetes**			
Known	1.38	0.15	0.89, 2.15
Undiagnosed	0.86	0.61	0.48, 1.55
**Hypertension**			
Known	1.37	0.18	0.86, 2.17
Undiagnosed	0.98	0.94	0.53, 1.82
**Dyslipidemia**			
Known	1.21	0.57	0.62, 2.38
Undiagnosed	1.24	0.31	0.82, 1.88
**TOAST**			
Small Vessel Disease	0.36	0.02 *	0.15, 0.84
Large Vessel Disease	0.67	0.37	0.28, 1.61
Cardioembolic Stroke	0.66	0.37	0.27, 1.63
Stroke of Determined Etiology	0.37	0.08	0.12, 1.14
(b)			
**Age**			
>60	1.22	0.40	0.77, 1.95
45–60	1.26	0.30	0.81, 1.95
<45	-	-	-
**Gender**			
Male	0.88	0.57	0.57, 1.36
Female	-	-	-
**Smoking**			
Yes	0.70	0.03 *	0.52, 0.96
**NIHSS Admission**			
5–10	5.31	<0.001 *	4.00, 7.05
11–40	16.57	<0.001 *	11.48, 23.93
**Diabetes**			
Known	1.62	<0.001 *	1.20, 2.18
Undiagnosed	1.49	0.047 *	1.01, 2.20
**Hypertension**			
Known	1.34	0.07	0.98, 1.84
Undiagnosed	1.23	0.31	0.82, 1.85
**Dyslipidemia**			
Known	0.85	0.42	0.56, 1.28
Undiagnosed	0.87	0.36	0.65, 1.17
**TOAST**			
Small Vessel Disease	0.50	0.03 *	0.27, 0.92
Large Vessel Disease	0.95	0.88	0.51, 1.78
Cardioembolic Stroke	0.61	0.13	0.32, 1.16
Stroke of Determined Etiology	0.50	0.06	0.24, 1.02
(c)			
**Age**			
>60	0.68	0.53	0.21, 2.24
45–60	1.19	0.56	0.66, 2.15
<45	-	-	-
**Gender**			
Male	2.52	0.50	0.18, 35.86
Female	-	-	-
**Smoking**			
Yes	0.53	0.06	0.28, 1.02
**NIHSS Admission**			
5–10	7.35	<0.001 *	4.10, 13.18
11–40	23.06	<0.001 *	10.87, 48.91
**Diabetes**			
Known	1.61	0.14	0.85, 3.03
Undiagnosed	1.11	0.78	0.56, 2.20
**Hypertension**			
Known	1.62	0.11	0.90, 2.94
Undiagnosed	1.06	0.90	0.49, 2.25
**Dyslipidemia**			
Known	0.90	0.85	0.31, 2.60
Undiagnosed	0.97	0.93	0.54, 1.74
**TOAST**			
Small Vessel Disease	0.61	0.35	0.21, 1.73
Large Vessel Disease	0.92	0.88	0.32, 2.71
Cardioembolic Stroke	0.89	0.84	0.29, 2.71
Stroke of Determined Etiology	0.32	0.10	0.08, 1.26
(d)			
**Age**			
>60	1.71	0.25	0.69, 4.24
45–60	1.01	0.98	0.39, 2.65
<45	-	-	-
**Gender**			
Male	0.97	0.91	0.52, 1.80
Female	-	-	-
**Smoking**			
Yes	0.69	0.22	0.38, 1.25
**NIHSS Admission**			
5–10	3.16	<0.001 *	1.91, 5.24
11–40	17.48	<0.001 *	8.54, 35.78
**Diabetes**			
Known	1.08	0.77	0.65, 1.79
Undiagnosed	1.58	0.31	0.65, 3.87
**Hypertension**			
Known	1.07	0.82	0.60, 1.90
Undiagnosed	0.49	0.13	0.20, 1.22
**Dyslipidemia**			
Known	1.13	0.66	0.65, 1.98
Undiagnosed	1.01	0.99	0.55, 1.83
**TOAST**			
Small Vessel Disease	0.64	0.38	0.23, 1.74
Large Vessel Disease	0.73	0.56	0.26, 2.09
Cardioembolic Stroke	1.00	0.99	0.35, 2.81
Stroke of Determined Etiology	0.27	0.044 *	0.07, 0.97
(e)			
**Age**			
>60	3.21	0.12	0.75, 13. 84
45–60	2.71	0.16	0.67, 10.99
<45	-	-	-
**Gender**			
Male	0.55	0.35	0.16, 1.90
Female	-	-	-
**Smoking**			
Yes	0.77	0.54	0.33, 1.79
**NIHSS Admission**			
5–10	3.10	0.006 *	1.38, 6.92
11–40	29.90	<0.001 *	10.27, 87.04
**Diabetes**			
Known	0.72	0.44	0.31, 1.67
Undiagnosed	2.18	0.15	0.76, 6.23
**Hypertension**			
Known	3.28	0.01 *	1.32, 8.14
Undiagnosed	3.57	0.03 *	1.12, 11.41
**Dyslipidemia**			
Known	0.24	0.07	0.05, 1.12
Undiagnosed	0.78	0.55	0.36, 1.73
**TOAST**			
Small Vessel Disease	2.52	0.49	0.18, 35.30
Large Vessel Disease	3.39	0.36	0.24, 47.27
Cardioembolic Stroke	2.06	0.61	0.13, 32.12
Stroke of Determined Etiology	1.55	0.77	0.09, 27.37

* *p* ≤ 0.05.

**Table 5:** (**a**) Multivariate bivariate logistic regression for factors associated with mortality at 90 days for Bangladeshis. (**b**) Multivariate bivariate logistic regression for factors associated with mortality at 90 days for Indians. (**c**) Multivariate bivariate logistic regression for factors associated with mortality at 90 days for Nepalese. (**d**) Multivariate bivariate logistic regression for factors associated with mortality at 90 days for Pakistanis. (**e**) Multivariate bivariate logistic regression for factors associated with mortality at 90 days for Sri Lankans.

Variable	aOR	*p*-Value	95% CI
(a)			
**Age**			
>60	3.36	0.13	0.69, 16.30
45–60	3.31	0.09	0.83, 13.25
<45	-	-	-
**Gender**			
Male	0.37	0.32	0.05, 2.65
Female	-	-	-
**Smoking**			
Yes	0.38	0.10	0.12, 1.20
**NIHSS Admission**			
5–10	2.30	0.32	0.44, 12.04
11–40	19.75	<0.001 *	5.58, 69.87
**Diabetes**			
Known	1.02	0.96	0.40, 2.61
Undiagnosed	0.78	0.71	0.20, 2.95
**Hypertension**			
Known	0.60	0.30	0.23, 1.57
Undiagnosed	0.33	0.14	0.08, 1.43
**Dyslipidemia**			
Known	0.32	0.30	0.04, 2.76
Undiagnosed	0.37	0.07	0.13, 1.08
**TOAST**			
Small Vessel Disease	-	-	-
Large Vessel Disease	0.45	0.32	0.09, 2.17
Cardioembolic Stroke	1.29	0.73	0.29, 5.72
Stroke of Determined Etiology	0.94	0.95	0.15, 5.85
(b)			
**Age**			
>60	2.30	0.31	0.47, 11.30
45–60	2.65	0.21	0.58, 12.18
<45	-	-	-
**Gender**			
Male	0.56	0.22	0.23, 1.39
Female	-	-	-
**Smoking**			
Yes	0.18	0.02 *	0.04, 0.78
**NIHSS Admission**			
5–10	1.60	0.31	0.64, 3.97
11–40	5.85	<0.001 *	2.70, 12.69
**Diabetes**			
Known	1.34	0.44	0.64, 2.79
Undiagnosed	1.06	0.92	0.36, 3.11
**Hypertension**			
Known	1.90	0.11	0.86, 4.19
Undiagnosed	0.46	0.33	0.10, 2.20
**Dyslipidemia**			
Known	0.53	0.21	0.20, 1.41
Undiagnosed	0.54	0.17	0.23, 1.29
**TOAST**			
Small Vessel Disease	0.15	0.02 *	0.03, 0.76
Large Vessel Disease	1.03	0.97	0.27, 3.95
Cardioembolic Stroke	0.93	0.91	0.24, 3.60
Stroke of Determined Etiology	0.40	0.30	0.07, 2.26
(c)			
**Age**			
>60	0.12	0.29	0.002, 6.22
45–60	0.22	0.21	0.02, 2.32
<45	-	-	-
**Gender**			
Male	-	-	-
Female	-	-	-
**Smoking**			
Yes	-	-	-
**NIHSS Admission**			
5–10	-	-	-
11–40	26.19	0.044 *	1.09, 628.9
**Diabetes**			
Known	53.5	0.006 *	3.16, 904.34
Undiagnosed	-	-	-
**Hypertension**			
Known	3.94	0.26	0.35, 43.79
Undiagnosed	-	-	-
**Dyslipidemia**			
Known	3.08	0.51	0.11, 86.82
Undiagnosed	-	-	-
**TOAST**			
Small Vessel Disease	0.15	0.39	0.002, 11.00
Large Vessel Disease	0.26	0.50	0.10, 13.00
Cardioembolic Stroke	0.39	0.62	0.10, 15.94
Stroke of Determined Etiology	-	-	-
(d)			
**Age**			
>60	0.76	0.71	0.17, 3.30
45–60	0.26	0.13	0.04, 1.51
<45	-	-	-
**Gender**			
Male	1.05	0.95	0.29, 3.79
Female	-	-	-
**Smoking**			
Yes	2.32	0.16	0.71, 7.54
**NIHSS Admission**			
5–10	1.52	0.57	0.36, 6.45
11–40	15.59	<0.001 *	5.19, 46.85
**Diabetes**			
Known	1.58	0.38	0.57, 4.41
Undiagnosed	0.45	0.51	0.04, 4.97
**Hypertension**			
Known	0.69	0.54	0.21, 2.25
Undiagnosed	0.20	0.17	0.02, 2.04
**Dyslipidemia**			
Known	0.69	0.55	0.21, 2.28
Undiagnosed	1.18	0.80	0.32, 4.30
**TOAST**			
Small Vessel Disease	-	-	-
Large Vessel Disease	2.16	0.31	0.49, 9.47
Cardioembolic Stroke	4.86	0.03 *	1.21, 19.46
Stroke of Determined Etiology	-	-	-
(e)			
**Age**			
>60	2.28	0.55	0.15, 33.59
45–60	0.82	0.88	0.07, 10.17
<45	-	-	-
**Gender**			
Male	0.53	0.62	0.04, 6.62
Female	-	-	-
**Smoking**			
Yes	0.19	0.19	0.02, 2.26
**NIHSS Admission**			
5–10	1.02	0.99	0.08, 13.44
11–40	14.00	0.008 *	1.98, 99.09
**Diabetes**			
Known	1.93	0.58	0.19, 19.58
Undiagnosed	1.39	0.79	0.13, 14.64
**Hypertension**			
Known	0.66	0.71	0.08, 5.72
Undiagnosed	-	-	-
**Dyslipidemia**			
Known	1.20	0.90	0.08, 18.65
Undiagnosed	0.34	0.32	0.04, 2.86
**TOAST**			
Small Vessel Disease	-	-	-
Large Vessel Disease	4.97	0.23	0.36, 68.85
Cardioembolic Stroke	6.31	0.16	0.47, 84.61
Stroke of Determined Etiology	-	-	-

* *p* ≤ 0.05.

## Data Availability

Data is contained within the article and Supplementary Materials.

## Supplementary Materials

The following supporting information can be downloaded at: https://www.mdpi.com/article/10.3390/neurolint17090140/s1, Figure S1: Multivariable bivariate logistic regression analysis of the risk factors associated with a higher mRS score (3–6) at 90 days; Figure S2: Multivariable bivariate logistic regression analysis of the risk factors associated with mortality at 90 day title; Table S1: Baseline characteristics excluding stroke mimics; Table S2: Values reported are χ^2^ adjusted residuals; Table S3: Multivariable multiple regression for mortality at 90 days and mRS at 90 days; Table S4: Multivariate bivariate logistic regression for factors associated with a poor functional outcome (mRS of 3–6) at 90 days; Table S5: Multivariate bivariate logistic regression for factors associated with mortality at 90 days.
