# Supplementary material for: Epidemiological and Clinical Characteristics of Acute Stroke in a Multi-Ethnic South Asian Population
Source: Neurol Int. 2025 Sep 5;17(9):140. doi: 10.3390/neurolint17090140 (PMC12472970; doi:10.3390/neurolint17090140)
Supplement: Supplementary file 1 [file neurolint-17-00140-s001.zip › neurolint-3767607-supplementary/neurolint-3767607 Supplementary/Asian Population Supplementary Data.pdf]

	Overall	Bangladesh	India	Nepal	Pakistan	Sri Lanka	Overall Significance
**Total patients**	6948	1584	3080	976	842	466	
**Age**	55.0 (48.0–63.0)	54.0 (47.0–62.0)	56.0 (49.0–63.0)	50.0 (44.0–55.0)	65.0 (55.0–74.0)	56.0 (49.0–62.0)	<.001*
<45	1075 (15.5)	265 (16.7)	415 (13.5)	260 (26.6)	85 (10.1)	50 (10.7)	
45-60	3650 (52.5)	859 (54.2)	1641 (53.3)	658 (67.4)	222 (26.4)	270 (57.9)	
>60	2223 (32.0)	460 (29.0)	1024 (33.2)	58 (5.9)	535 (63.5)	146 (31.3)	
**Gender**							<.001
Male	6468 (93.1)	1546 (97.6)	2828 (91.8)	963 (98.7)	704 (83.6)	427 (91.6)	
Female	480 (6.9)	38 (2.4)	252 (8.2)	13 (1.3)	138 (16.4)	39 (8.4)	
**Stroke Type**							<.001
Central Venous Sinus Thrombosis	127 (1.8)	23 (1.5)	54 (1.8)	24 (2.5)	19 (2.3)	7 (1.5)	
Intracerebral Hemorrhage	1101 (15.8)	295 (18.6)	392 (12.7)	256 (26.2)	90 (10.7)	68 (14.6)	
Ischemic Stroke	5070 (73.0)	1168 (73.7)	2273 (73.8)	642 (65.8)	629 (74.7)	358 (76.8)	
Transient Ischemic Attack	650 (9.4)	98 (6.2)	361 (11.7)	54 (5.5)	104 (12.4)	33 (7.1)	
**TOAST**							0.04
Small vessel disease	2398 (46.3)	573 (48.4)	1069 (46.1)	320 (48.3)	265 (40.9)	171 (47.0)	
Large vessel disease	1128 (21.8)	256 (21.6)	505 (21.8)	138 (20.8)	146 (22.5)	83 (22.8)	
Cardioembolic	951 (18.4)	218 (18.4)	425 (18.3)	105 (15.8)	142 (21.9)	61 (16.8)	
Determined aetiology	447 (8.6)	77 (6.5)	216 (9.3)	57 (8.6)	63 (9.7)	34 (9.3)	
Undetermined aetiology	255 (4.9)	60 (5.1)	105 (4.5)	43 (6.5)	32 (4.9)	15 (4.1)	
**Medical comorbidities**							
Diabetes	2610 (37.6)	664 (41.9)	1196 (38.8)	173 (17.7)	413 (49.0)	164 (35.2)	<.001
Hypertension	3780 (54.4)	892 (56.3)	1653 (53.7)	448 (45.9)	535 (63.5)	252 (54.1)	<.001
Dyslipidaemia	807 (11.7)	180 (11.4)	361 (11.8)	39 (4.0)	174 (20.7)	53 (11.4)	<.001
Smoking	1604 (23.2)	436 (27.6)	727 (23.7)	179 (18.5)	142 (16.9)	120 (25.9)	<.001
Obesity (BMI ≥ 30)	1077 (15.6)	167 (10.6)	475 (15.5)	150 (15.4)	228 (27.3)	57 (12.3)	<.001
**Management**							
Thrombolysis	616 (8.9)	125 (7.9)	292 (9.5)	84 (8.6)	79 (9.4)	36 (7.7)	0.35
Thrombectomy	240 (3.5)	46 (2.9)	110 (3.6)	44 (4.5)	22 (2.6)	18 (3.9)	0.15
**NIHSS Admission**							<.001
Mild Stroke (0-4)	4030 (58.3)	884 (56.2)	1877 (61.2)	470 (48.3)	526 (62.7)	273 (59.1)	
Moderate Stroke (5-10)	1561 (22.6)	355 (22.6)	694 (22.6)	239 (24.5)	174 (20.7)	99 (21.4)	

	Overall	Bangladesh	India	Nepal	Pakistan	Sri Lanka	Overall Significance
Severe Stroke (≥11)	1326 (19.2)	335 (21.3)	497 (16.2)	265 (27.2)	139 (16.6)	90 (19.5)	
**mRS at admission**							<.001
0-2	6818 (98.1)	1565 (98.8)	3033 (98.5)	975 (99.9)	779 (92.5)	466 (100.0)	
3-6	129 (1.9)	19 (1.2)	46 (1.5)	1 (0.1)	63 (7.5)	0 (0)	
**NIHSS at discharge**							<.001
Mild Stroke (0-4)	3705 (66.6)	810 (62.4)	1721 (69.3)	452 (58.0)	473 (73.3)	249 (69.7)	
Moderate Stroke (5-10)	1117 (20.1)	300 (23.1)	463 (18.7)	187 (24.0)	99 (15.3)	68 (19.0)	
Severe Stroke (≥11)	740 (13.3)	189 (14.5)	298 (12.0)	140 (18.0)	73 (11.3)	40 (11.2)	
**mRS at 90 days**							<.001
0-2	3553 (70.1)	788 (68.5)	1657 (73.4)	437 (64.0)	433 (67.8)	238 (71.0)	
3-6	1513 (29.9)	362 (31.5)	602 (26.6)	246 (36.0)	206 (32.2)	97 (29.0)	
**Mortality at 90 days**							0.02
No	4834 (95.4)	1085 (94.3)	2178 (96.4)	654 (95.8)	602 (94.2)	315 (94.0)	
Yes	232 (4.6)	65 (5.7)	81 (3.6)	29 (4.2)	37 (5.8)	20 (6.0)	

Supplementary Table S1. Baseline characteristics excluding stroke mimics

*Bonferroni correction was applied using Dunn's test. All other p-values reported are unadjusted p-values derived from χ^2 test
