# Supplementary material for: Epidemiological and Clinical Characteristics of Acute Stroke in a Multi-Ethnic South Asian Population
Source: Neurol Int. 2025 Sep 5;17(9):140. doi: 10.3390/neurolint17090140 (PMC12472970; doi:10.3390/neurolint17090140)
Supplement: Supplementary file 1 [file neurolint-17-00140-s001.zip › neurolint-3767607-supplementary/neurolint-3767607 Supplementary/Asian Subpopulation Figures Supplementary.pdf]

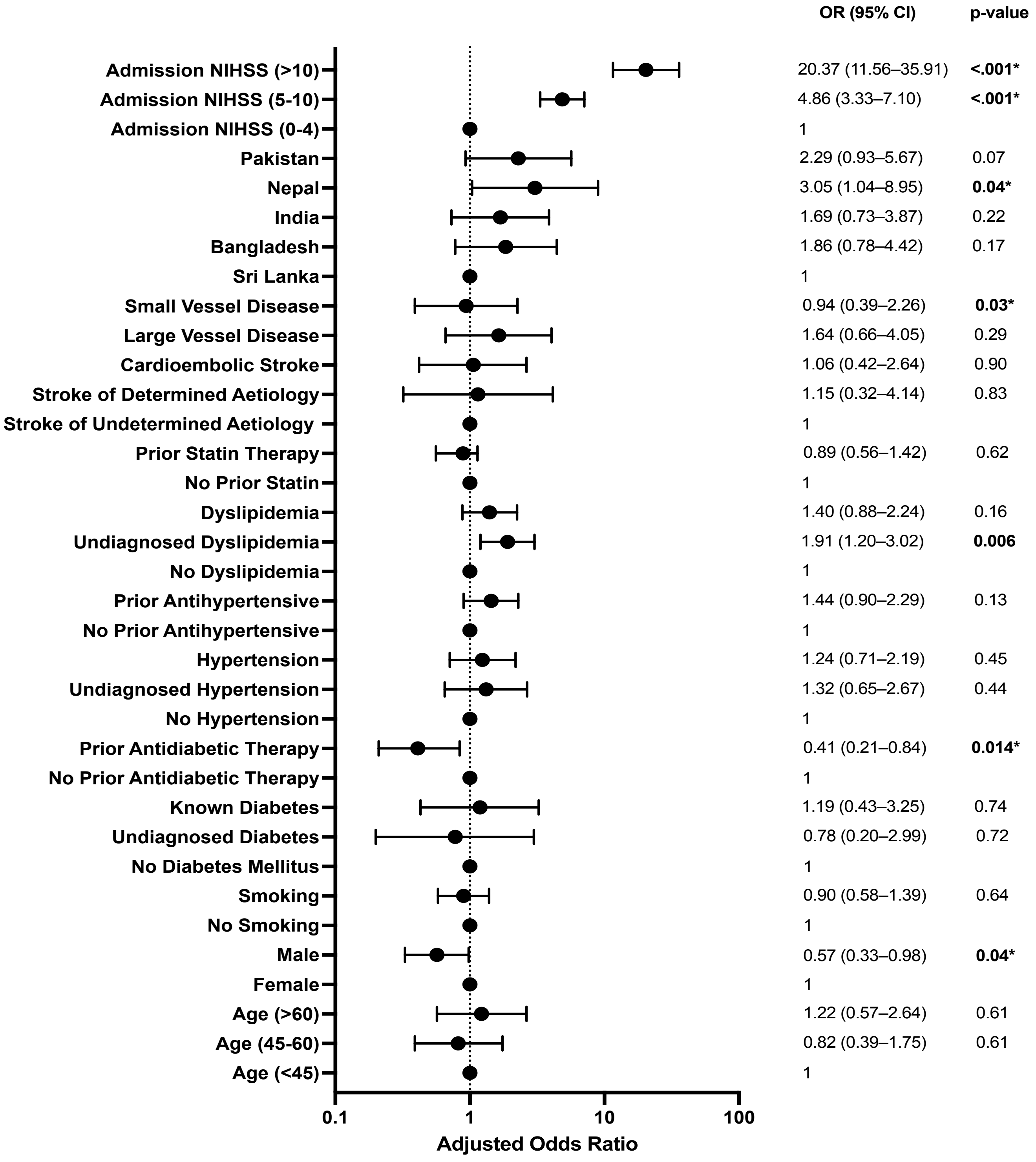

$^*p \leq 0.05$

Supplementary Figure S1. Multivariable bivariate logistic regression analysis of the risk factors associated with a higher mRS score (3–6) at 90 days

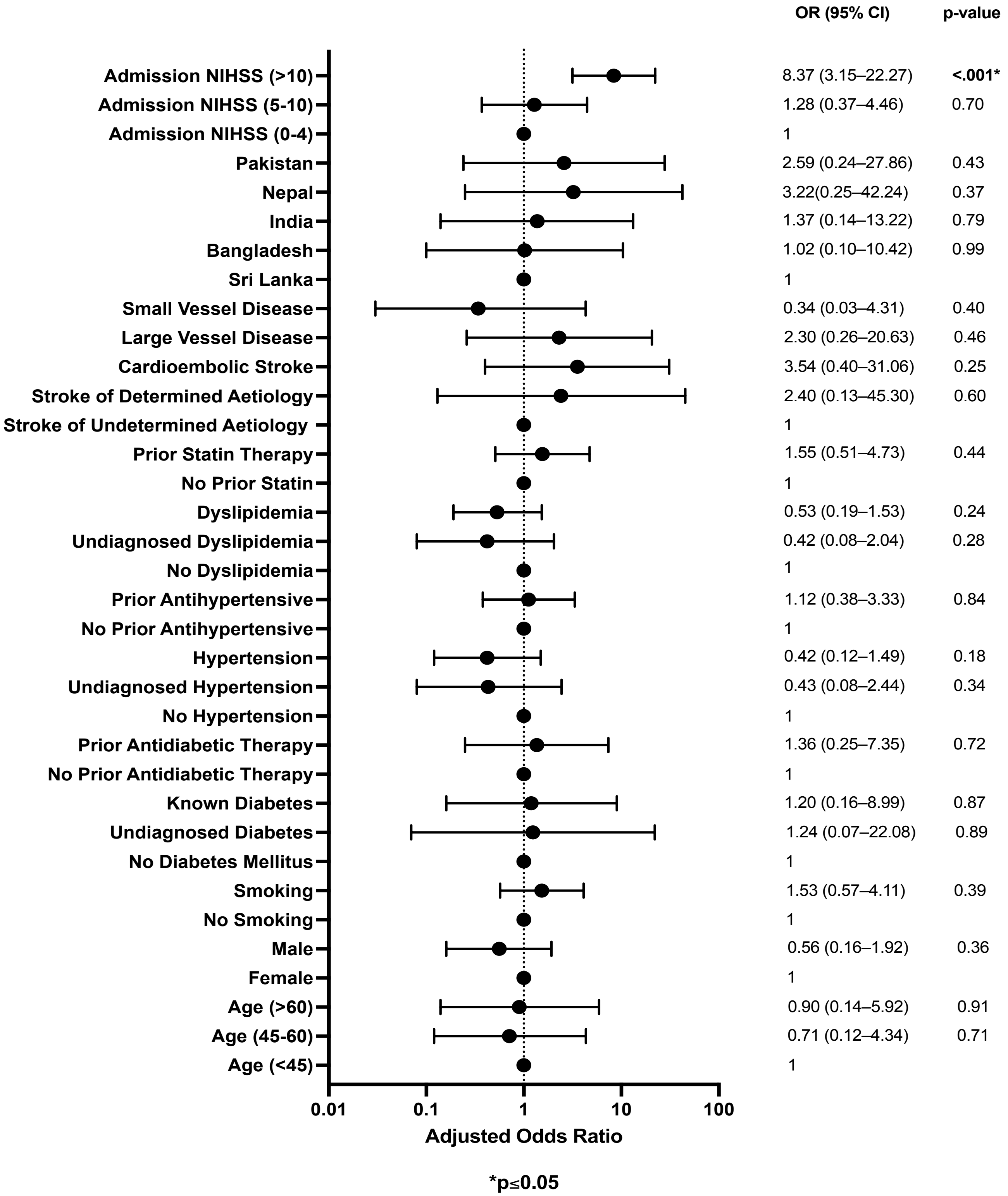

Supplemetary Figure S2. Multivariable bivariate logistic regression analysis of the risk factors associated with mortality at 90 days
