# Supplementary material for: Epidemiological and Clinical Characteristics of Acute Stroke in a Multi-Ethnic South Asian Population
Source: Neurol Int. 2025 Sep 5;17(9):140. doi: 10.3390/neurolint17090140 (PMC12472970; doi:10.3390/neurolint17090140)
Supplement: Supplementary file 1 [file neurolint-17-00140-s001.zip › neurolint-3767607-supplementary/neurolint-3767607 Supplementary/Chi Square Asian Without Mimics.pdf]

	Bangladesh	India	Nepal	Pakistan	Sri Lanka
Age					
<45	1.58	**-4.11***	**10.41***	**-4.60***	-2.93
45-60	1.54	1.11	**10.04***	**-16.22***	2.42
>60	-2.87	2.00	**-18.82***	**20.93***	-0.32
Gender					
Male	**8.05***	**-3.73***	**7.41***	**-11.57***	-1.29
Female	**-8.05***	**3.73***	**-7.41***	**11.57***	1.29
TOAST					
Small vessel disease	1.64	-0.29	1.09	-2.95	0.27
Large vessel disease	-0.15	-0.02	-0.65	0.49	0.49
Cardioembolic	0.05	-0.07	-1.80	2.50	-0.82
Determined aetiology	-2.97	1.57	-0.03	1.06	0.50
Undetermined aetiology	0.26	-1.19	1.99	0.02	-0.73
Stroke Subtypes					
Central Venous Sinus Thrombosis	-1.27	-0.41	1.59	0.99	-0.54
Intracerebral Hemorrhage	**3.45***	**-6.35***	**9.58***	**-4.37***	-0.77
Ischemic Stroke	0.78	1.39	**-5.46***	1.21	1.94
Transient Ischemic Attack	**-4.93***	**6.04***	**-4.42***	3.18	-1.75
Medical Comorbidities					
Diabetes					
Known	**4.07***	1.95	**-13.80***	**7.34***	-1.09
Undiagnosed	1.38	0.57	0.70	**-3.83***	0.57
No	**-4.84***	-2.25	**12.92***	**-4.64***	0.69
Hypertension					
Known	1.76	-1.11	**-5.76***	**5.67***	-0.15
Undiagnosed	-1.05	0.38	**6.07***	**-4.35***	-1.75
No	-1.08	0.91	1.42	-2.70	1.56
Dyslipidaemia					

Known	-0.38	0.24	**-7.99***	**8.74***	-0.15
Undiagnosed	0.31	1.75	0.28	**-6.13***	**3.62***
No	-0.03	-1.77	**4.98***	-0.10	**-3.23***
Smoking					
Yes	**4.75***	0.91	**-3.78***	**-4.58**	1.43
No	**-4.75***	-0.91	**3.78***	**4.58**	-1.43
BMI					
<30	**6.23***	0.18	0.12	**-9.97***	2.00
≥30	**-6.23***	-0.18	-0.12	**9.97***	-2.00
mRS at admission					
0-2	2.21	2.00	**4.38***	**-12.90***	**3.07***
3-6	-2.21	-2.00	**-4.38***	**12.90***	**-3.07***
NIHSS Admission					
0-4	-1.92	**4.39***	**-6.83***	**2.78***	0.37
5-10	-0.01	0.09	1.59	-1.35	-0.61
≥11	2.42	**-5.60***	**6.87***	-2.04	0.18
Mortality at 90 days					
Yes	1.98	**-3.04***	-0.45	1.57	1.26
No	-1.98	**3.04***	0.45	-1.57	-1.26
mRS at 90 days					
0-2	-1.36	**4.49***	**-3.78***	-1.40	0.38
3-6	1.36	**-4.49***	**3.78***	1.40	-0.38
NIHSS at discharge					
0-4	**-3.72***	**3.87***	**-5.48***	**3.85***	1.30
5-10	**3.10***	-2.39	**2.95***	**-3.19***	-0.50
≥11	1.51	-2.56	**4.14***	-1.58	-1.21

Supplementary Table S2. Values reported are χ^2 adjusted residuals. *p≤0.05 with Bonferroni correction

Supplementary Table S3. Multivariable multiple regression for mortality at 90 days and mRS at 90 days

mRS at 90 days	Coef.	Std. Err.	t-value	95% CI	p-value
**Age**					
45-60	-0.021	0.059	-0.35	(-0.14, 0.096)	0.72
>60	0.037	0.061	0.61	(-0.08, 0.157)	0.55
**Gender**					
Male	-0.098	0.046	-2.11	(-0.19, -0.007)	**0.04***
**Ethnicity**					
India	-0.016	0.034	-0.48	(-0.08, 0.051)	0.64
Nepal	0.077	0.066	1.17	(-0.05, 0.206)	0.24
Pakistan	0.034	0.046	0.75	(-0.06, 0.124)	0.46
Sri Lanka	-0.081	0.061	-1.33	(-0.20, 0.038)	0.18
**Smoking**					
Yes	-0.015	0.033	-0.45	(-0.08, 0.050)	0.65
**Diabetes**					
Known	0.045	0.082	0.55	(-0.12, 0.205)	0.58
Undiagnosed	-0.019	0.103	-0.19	(-0.22, 0.183)	0.85
**Hypertension**					
Known	0.025	0.042	0.60	(-0.06, 0.108)	0.55
Undiagnosed					0.53
**Dyslipidaemia**					
Known	0.056	0.037	1.50	(-0.02, 0.129)	0.14
Undiagnosed	0.103	0.037	2.80	(0.03, 0.175)	**0.005***
**NIHSS Admission**					
5-10	0.293	0.033	8.87	(0.23, 0.357)	**<.001***
11-40	0.603	0.045	13.5	(0.52, 0.691)	**<.001***
**Diabetic Therapy**					
Yes	-0.143	0.058	-2.47	(-0.26, -0.029)	**0.014***

**Statin**					
Yes	-0.016	0.037	-0.44	(-0.09, 0.057)	0.66
**Antihypertensive**					
Yes	0.059	0.037	1.61	(-0.01, 0.131)	0.11
**Mortality at 90 days**	**Coef.**	**Std. Err.**	**t-value**	**95% CI**	**p-value**
**Age**					
45-60	-0.006	0.026	-0.23	(-0.06, 0.045)	0.82
>60	0.002	0.027	0.08	(-0.05, 0.054)	0.94
**Gender**					
Male	-0.025	0.020	-1.23	(-0.06, 0.015)	0.22
**Ethnicity**					
India	0.005	0.015	0.34	(-0.02, 0.034)	0.73
Nepal	0.041	0.029	1.42	(-0.02, 0.097)	0.16
Pakistan	0.027	0.020	1.38	(-0.01, 0.066)	0.17
Sri Lanka	0.005	0.026	0.19	(-0.05, 0.057)	0.85
**Smoking**					
Yes	0.012	0.015	0.85	(-0.02, 0.041)	0.40
**Diabetes**					
Known	-0.007	0.035	-0.19	(-0.08, 0.063)	0.85
Undiagnosed	0.009	0.045	0.19	(-0.08, 0.097)	0.85
**Hypertension**					
Known	-0.016	0.018	-0.87	(-0.05, 0.020)	0.38
Undiagnosed	-0.021	0.024	-0.88	(-0.07, 0.026)	0.38
**Dyslipidaemia**					
Known	-0.019	0.016	-1.15	(-0.05, 0.013)	0.25
Undiagnosed	-0.013	0.016	-0.82	(-0.04, 0.018)	0.41
**NIHSS Admission**					
5-10	0.001	0.014	0.10	(-0.03, 0.030)	0.92
11-40	0.125	0.019	6.44	(0.09, 0.164)	**<.001***
**Diabetic Therapy**					

Yes	0.019	0.025	0.77	(-0.03, 0.069)	0.44
**Statin**					
Yes	0.016	0.016	0.96	(-0.02, 0.048)	0.34
**Antihypertensive**					
Yes	0.001	0.016	0.08	(-0.03, 0.033)	0.94
**mRS at 90 days**					
**Obs**		879	**R-sq**		0.272
**Parms**		24	**F**		13.92
**Root-mean-square deviation**		0.396	**Prob > F**		<.001
**Mortality at 90 days**					
**Obs**		879	**R-sq**		0.091
**Parms**		24	**F**		3.70
**Root-mean-square deviation**		0.173	**Prob > F**		<.001

*p≤0.05

Variable	aOR	p-value	95% CI
**Age**			
>60	0.73	0.34	0.38, 1.40
45-60	0.75	0.35	0.42, 1.36
<45	-	-	-
**Gender**			
Male	1.23	0.76	0.34, 4.40
Female	-	-	-
**Smoking**			
Yes	1.21	0.36	0.80, 1.82
**NIHSS Admission**			
5-10	5.35	**<.001***	3.49, 8.22
11-40	30.67	**<.001***	17.60, 53.42
**Diabetes**			
Known	1.38	0.15	0.89, 2.15
Undiagnosed	0.86	0.61	0.48, 1.55
**Hypertension**			
Known	1.37	0.18	0.86, 2.17
Undiagnosed	0.98	0.94	0.53, 1.82
**Dyslipidaemia**			
Known	1.21	0.57	0.62, 2.38
Undiagnosed	1.24	0.31	0.82, 1.88
**TOAST**			
Small Vessel Disease	0.36	**0.02***	0.15, 0.84
Large Vessel Disease	0.67	0.37	0.28, 1.61
Cardioembolic Stroke	0.66	0.37	0.27, 1.63
Stroke of Determined Etiology	0.37	0.08	0.12, 1.14

Supplementary Table S4. i) Multivariate bivariate logistic regression for factors associated with a poor functional outcome (mRS of 3–6) at 90 days for Bangladeshis.

*p≤0.05

Variable	aOR	p-value	95% CI
**Age**			
>60	1.22	0.40	0.77, 1.95
45-60	1.26	0.30	0.81, 1.95
<45	-	-	-
**Gender**			
Male	0.88	0.57	0.57, 1.36
Female	-	-	-
**Smoking**			
Yes	0.70	**0.03***	0.52, 0.96
**NIHSS Admission**			
5-10	5.31	**<.001***	4.00, 7.05
11-40	16.57	**<.001***	11.48, 23.93
**Diabetes**			
Known	1.62	**<.001***	1.20, 2.18
Undiagnosed	1.49	**0.047***	1.01, 2.20
**Hypertension**			
Known	1.34	0.07	0.98, 1.84
Undiagnosed	1.23	0.31	0.82, 1.85
**Dyslipidaemia**			
Known	0.85	0.42	0.56, 1.28
Undiagnosed	0.87	0.36	0.65, 1.17
**TOAST**			
Small Vessel Disease	0.50	**0.03***	0.27, 0.92
Large Vessel Disease	0.95	0.88	0.51, 1.78
Cardioembolic Stroke	0.61	0.13	0.32, 1.16
Stroke of Determined Etiology	0.50	0.06	0.24, 1.02

Supplementary Table S4. ii) Multivariate bivariate logistic regression for factors associated with a poor functional outcome (mRS of 3–6) at 90 days for Indians.

*p≤0.05

Variable	aOR	p-value	95% CI
**Age**			
>60	0.68	0.53	0.21, 2.24
45-60	1.19	0.56	0.66, 2.15
<45	-	-	-
**Gender**			
Male	2.52	0.50	0.18, 35.86
Female	-	-	-
**Smoking**			
Yes	0.53	0.06	0.28, 1.02
**NIHSS Admission**			
5-10	7.32	**<.001***	4.08, 13.11
11-40	22.94	**<.001***	10.82, 48.66
**Diabetes**			
Known	1.60	0.15	0.85, 3.02
Undiagnosed	1.10	0.78	0.55, 2.20
**Hypertension**			
Known	1.62	0.11	0.90, 2.94
Undiagnosed	1.06	0.90	0.49, 2.25
**Dyslipidaemia**			
Known	0.90	0.85	0.31, 2.60
Undiagnosed	0.97	0.93	0.54, 1.74
**TOAST**			
Small Vessel Disease	0.61	0.35	0.21, 1.73
Large Vessel Disease	0.92	0.88	0.32, 2.70
Cardioembolic Stroke	0.89	0.84	0.29, 2.71
Stroke of Determined Etiology	0.32	0.10	0.08, 1.26

Supplementary Table S4. iii) Multivariate bivariate logistic regression for factors associated with a poor functional outcome (mRS of 3–6) at 90 days for Nepalese.

*p≤0.05

Variable	aOR	p-value	95% CI
**Age**			
>60	1.71	0.25	0.69, 4.24
45-60	1.01	0.98	0.39, 2.65
<45	-	-	-
**Gender**			
Male	0.97	0.91	0.52, 1.80
Female	-	-	-
**Smoking**			
Yes	0.69	0.22	0.38, 1.25
**NIHSS Admission**			
5-10	3.16	**<.001***	1.91, 5.24
11-40	17.48	**<.001***	8.54, 35.78
**Diabetes**			
Known	1.08	0.77	0.65, 1.79
Undiagnosed	1.58	0.31	0.65, 3.87
**Hypertension**			
Known	1.07	0.82	0.60, 1.90
Undiagnosed	0.49	0.13	0.20, 1.22
**Dyslipidaemia**			
Known	1.13	0.66	0.65, 1.98
Undiagnosed	1.01	0.99	0.55, 1.83
**TOAST**			
Small Vessel Disease	0.64	0.38	0.23, 1.74
Large Vessel Disease	0.73	0.56	0.26, 2.09
Cardioembolic Stroke	1.00	0.99	0.35, 2.81
Stroke of Determined Etiology	0.27	**0.044***	0.07, 0.97

Supplementary Table S4. iv) Multivariate bivariate logistic regression for factors associated with a poor functional outcome (mRS of 3–6) at 90 days for Pakistanis.

*p≤0.05

Variable	aOR	p-value	95% CI
**Age**			
>60	3.21	0.12	0.75, 13.84
45-60	2.71	0.16	0.67, 10.99
<45	-	-	-
**Gender**			
Male	0.55	0.35	0.16, 1.90
Female	-	-	-
**Smoking**			
Yes	0.77	0.54	0.33, 1.79
**NIHSS Admission**			
5-10	3.10	**0.006***	1.38, 6.92
11-40	29.90	**<.001***	10.27, 87.04
**Diabetes**			
Known	0.72	0.44	0.31, 1.67
Undiagnosed	2.18	0.15	0.76, 6.23
**Hypertension**			
Known	3.28	**0.01***	1.32, 8.14
Undiagnosed	3.57	**0.03***	1.12, 11.41
**Dyslipidaemia**			
Known	0.24	0.07	0.05, 1.12
Undiagnosed	0.78	0.55	0.36, 1.73
**TOAST**			
Small Vessel Disease	2.52	0.49	0.18, 35.30
Large Vessel Disease	3.39	0.36	0.24, 47.27
Cardioembolic Stroke	2.06	0.61	0.13, 32.12
Stroke of Determined Etiology	1.55	0.77	0.09, 27.37

Supplementary Table S4. v) Multivariate bivariate logistic regression for factors associated with a poor functional outcome (mRS of 3–6) at 90 days for Sri Lankans.

*p≤0.05

Variable	aOR	p-value	95% CI
**Age**			
>60	3.36	0.13	0.69, 16.30
45-60	3.31	0.09	0.83, 13.25
<45	-	-	-
**Gender**			
Male	0.37	0.32	0.05, 2.65
Female	-	-	-
**Smoking**			
Yes	0.38	0.10	0.12, 1.20
**NIHSS Admission**			
5-10	2.30	0.32	0.44, 12.04
11-40	19.75	**<.001***	5.58, 69.87
**Diabetes**			
Known	1.02	0.96	0.40, 2.61
Undiagnosed	0.78	0.71	0.20, 2.95
**Hypertension**			
Known	0.60	0.30	0.23, 1.57
Undiagnosed	0.33	0.14	0.08, 1.43
**Dyslipidaemia**			
Known	0.32	0.30	0.04, 2.76
Undiagnosed	0.37	0.07	0.13, 1.08
**TOAST**			
Small Vessel Disease	-	-	-
Large Vessel Disease	0.45	0.32	0.09, 2.17
Cardioembolic Stroke	1.29	0.73	0.29, 5.72
Stroke of Determined Etiology	0.94	0.95	0.15, 5.85

Supplementary Table S5. i) Multivariate bivariate logistic regression for factors associated with mortality at 90 days for Bangladeshis.

*p≤0.05

Variable	aOR	p-value	95% CI
**Age**			
>60	2.30	0.31	0.47, 11.30
45-60	2.65	0.21	0.58, 12.18
<45	-	-	-
**Gender**			
Male	0.56	0.22	0.23, 1.39
Female	-	-	-
**Smoking**			
Yes	0.18	**0.02***	0.04, 0.78
**NIHSS Admission**			
5-10	1.60	0.31	0.64, 3.97
11-40	5.85	**<.001***	2.70, 12.69
**Diabetes**			
Known	1.34	0.44	0.64, 2.79
Undiagnosed	1.06	0.92	0.36, 3.11
**Hypertension**			
Known	1.90	0.11	0.86, 4.19
Undiagnosed	0.46	0.33	0.10, 2.20
**Dyslipidaemia**			
Known	0.53	0.21	0.20, 1.41
Undiagnosed	0.54	0.17	0.23, 1.29
**TOAST**			
Small Vessel Disease	0.15	**0.02***	0.03, 0.76
Large Vessel Disease	1.03	0.97	0.27, 3.95
Cardioembolic Stroke	0.93	0.91	0.24, 3.60
Stroke of Determined Etiology	0.40	0.30	0.07, 2.26

Supplementary Table S5. ii) Multivariate bivariate logistic regression for factors associated with mortality at 90 days for Indians.

*p≤0.05

Variable	aOR	p-value	95% CI
**Age**			
>60	0.13	0.32	0.002, 7.41
45-60	0.21	0.20	0.02, 2.29
<45	-	-	-
**Gender**			
Male	-	-	-
Female	-	-	-
**Smoking**			
Yes	-	-	-
**NIHSS Admission**			
5-10	-	-	-
11-40	26.73	**0.043***	1.10, 646.88
**Diabetes**			
Known	51.92	**0.006***	3.10, 869.08
Undiagnosed	-	-	-
**Hypertension**			
Known	3.98	0.26	0.36, 43.97
Undiagnosed	-	-	-
**Dyslipidaemia**			
Known	3.35	0.49	0.11, 100.12
Undiagnosed	-	-	-
**TOAST**			
Small Vessel Disease	0.17	0.43	0.002, 14.16
Large Vessel Disease	0.27	0.52	0.10, 13.97
Cardioembolic Stroke	0.41	0.64	0.10, 17.31
Stroke of Determined Etiology	-	-	-

Supplementary Table S5. iii) Multivariate bivariate logistic regression for factors associated with mortality at 90 days for Nepalese.

*p≤0.05

Variable	aOR	p-value	95% CI
**Age**			
>60	0.76	0.71	0.17, 3.30
45-60	0.26	0.13	0.04, 1.51
<45	-	-	-
**Gender**			
Male	1.05	0.95	0.29, 3.79
Female	-	-	-
**Smoking**			
Yes	2.32	0.16	0.71, 7.54
**NIHSS Admission**			
5-10	1.52	0.57	0.36, 6.45
11-40	15.59	**<.001***	5.19, 46.85
**Diabetes**			
Known	1.58	0.38	0.57, 4.41
Undiagnosed	0.45	0.51	0.04, 4.97
**Hypertension**			
Known	0.69	0.54	0.21, 2.25
Undiagnosed	0.20	0.17	0.02, 2.04
**Dyslipidaemia**			
Known	0.69	0.55	0.21, 2.28
Undiagnosed	1.18	0.80	0.32, 4.30
**TOAST**			
Small Vessel Disease	-	-	-
Large Vessel Disease	2.16	0.31	0.49, 9.47
Cardioembolic Stroke	4.86	**0.03***	1.21, 19.46
Stroke of Determined Etiology	-	-	-

Supplementary Table S5. iv) Multivariate bivariate logistic regression for factors associated with mortality at 90 days for Pakistanis.

*p≤0.05

Variable	aOR	p-value	95% CI
**Age**			
>60	2.28	0.55	0.15, 33.59
45-60	0.82	0.88	0.07, 10.17
<45	-	-	-
**Gender**			
Male	0.53	0.62	0.04, 6.62
Female	-	-	-
**Smoking**			
Yes	0.19	0.19	0.02, 2.26
**NIHSS Admission**			
5-10	1.02	0.99	0.08, 13.44
11-40	14.00	**0.008***	1.98, 99.09
**Diabetes**			
Known	1.93	0.58	0.19, 19.58
Undiagnosed	1.39	0.79	0.13, 14.64
**Hypertension**			
Known	0.66	0.71	0.08, 5.72
Undiagnosed	-	-	-
**Dyslipidaemia**			
Known	1.20	0.90	0.08, 18.65
Undiagnosed	0.34	0.32	0.04, 2.86
**TOAST**			
Small Vessel Disease	-	-	-
Large Vessel Disease	4.97	0.23	0.36, 68.85
Cardioembolic Stroke	6.31	0.16	0.47, 84.61
Stroke of Determined Etiology	-	-	-

Supplementary Table S5. v) Multivariate bivariate logistic regression for factors associated with mortality at 90 days for Sri Lankans

*p≤0.05
